# Efficacy of N-acetylcysteine in Preventing Acute Kidney Injury and Major Adverse Cardiac Events After Cardiac Surgery: A Meta-Analysis and Trial Sequential Analysis

**DOI:** 10.3389/fmed.2022.795839

**Published:** 2022-06-22

**Authors:** Jingtao Zhao, Maowei Li, Chen Tan

**Affiliations:** ^1^Hebei Yanda Hospital, Langfang, China; ^2^No. 988th Hospital of Joint Logistic Support Force of PLA, Zhengzhou, China

**Keywords:** N-acetylcysteine, acute kidney injury, major adverse cardiac events (MACEs), cardiac surgery, trial sequential analysis

## Abstract

**Background:**

The effect of N-acetylcysteine (NAC), an antioxidant, on preventing acute kidney injury (AKI) and major adverse cardiac events (MACE) remains controversial. Therefore, we conducted this meta-analysis and trial sequential analysis to evaluate its efficacy on cardiac surgery-related adverse events.

**Methods:**

PubMed, Embase, and Cochrane Library were searched for relevant studies from inception to June 2021. We selected randomized controlled trials comparing NAC with controls in patients undergoing cardiac surgery.

**Results:**

Twenty-five studies including 2,444 patients met the inclusion criteria. The pooled results showed that there was no significant difference in the incidence of AKI between the NAC and control groups [relative risk (RR) = 0.91, 95% confidence interval (CI) = 0.77, 1.08, P = 0.28], but the trial sequential analysis (TSA) could not confirm this result. No difference was observed in the need for renal replacement therapy (RRT), all-cause mortality, MACE, length of stay in the intensive care unit (ICU), and length of stay in the hospital. Results of subgroup analysis results showed that intravenous infusion instead of oral NAC could significantly reduce the incidence of AKI and arrhythmia (RR = 0.84, 95% CI = 0.71, 0.99, *P* = 0.03, *I*^2^ = 3% and RR = 0.74, 95% CI = 0.61, 0.91, *P* = 0.004, *I*^2^ = 48%, respectively).

**Conclusion:**

Intravenous administration of NAC can reduce the incidence of AKI and arrhythmia in patients after cardiac surgery, but cannot reduce all-cause mortality, AMI, cardiac insufficiency, and the number of patients using RRT. Oral NAC has no significant effect on the outcomes of patients after cardiac surgery.

## Background

Coronary artery disease (CAD) is one of the major cardiovascular diseases worldwide and many CAD patients will be submitted for cardiac surgeries. The most common cardiac surgeries are coronary artery bypass graft (CABG) surgery and aortic or mitral valve repair or replacement (1, 2). They are commonly performed on-pump, which indicates that cardiopulmonary bypass (CPB) is used in the surgery. However, there is also a considerable number of cardiac surgeries that are performed off-pump without CPB assistance (3). Although the overall prognosis after cardiac surgery has been improved over the past decades, the occurrence of acute kidney injury (AKI) and major adverse cardiac events (MACEs) remains unsatisfactorily high (4, 5). AKI is the most common important complication in adult patients undergoing cardiac surgery and is associated with a prolonged hospital stay, use of dialysis, subsequent chronic kidney disease (CKD), and increased mortality (4). The incidence of AKI occurs in approximately 18% of patients undergoing cardiac surgery and approximately 2%-6% of them require renal replacement therapy (RRT). It is more likely for these kinds of patients to progress to CKD in the ensuing months and years than those who do not develop AKI and do not require RRT. A variety of risk factors, either renal or extrarenal, contribute to the development and progression of AKI after heart surgery, including renal ischemia, reperfusion, mechanical trauma, inflammation, hemolysis, oxidative stress, cholesterol emboli, and nephrotoxins (6). MACE is also an important composite primary endpoint assessed in most cardiovascular trials. Despite these unsatisfactory adverse events, cardiac surgery remains popular in CAD patients for its irreplaceable therapeutic effect. Thus, we must develop a more effective method to reduce the risk of postoperative complications of cardiac surgery.

N-acetylcysteine (NAC) is a cysteine prodrug and glutathione (GSH) precursor which has been used in clinical therapeutic practice as a mucolytic agent and for the treatment of numerous disorders including paracetamol intoxication, doxorubicin cardiotoxicity, ischemia-reperfusion cardiac injury and chemotherapy-induced toxicity associated with GSH deficiency for several decades (7). Also, NAC is a kind of free radical scavenger antioxidant agent and it is now well-known that it can reduce pro-inflammatory cytokines, oxygen free-radical production, and ameliorates ischemia-reperfusion injury which may consequently reduce postoperative complications in cardiac surgery (8). Researchers have investigated the efficacy and safety of NAC in several clinical trials in recent years. However, its effectiveness remains controversial. Although previous meta-analyses have investigated the role of NAC in preventing post-cardiac surgery complications, the results of them are conflicting (9–15). Therefore, we conducted this meta-analysis and trial sequential analysis (TSA) to further evaluate the efficacy of NAC in preventing AKI and MACEs after cardiac surgery.

## Methods

This meta-analysis adhered to the Preferred Reporting Items for Systematic Reviews and Meta-Analyses (PRISMA statement) guidelines (16).

### Data Sources and Search Strategy

Electronic databases including PubMed, Embase, and the Cochrane Library were systematically searched from inception to June 2021, using items related to “n-acetylcysteine,” and “cardiac surgery.” The search was limited to studies involving human subjects. No language restrictions or publication status were applied. The citations of included references were searched individually to identify potential additional relevant studies.

### Eligibility Criteria

The inclusion criteria were as follows: (1) study design: randomized controlled trials (RCTs); (2) population: patients undergoing cardiac surgery (>18 years old); (3) intervention: n-acetylcysteine (NAC) compared with placebo or standard of care; and (4) outcome: assessed at least one of the following outcomes: the incidence of acute kidney injury (AKI), the need for renal replacement therapy (RRT), all-cause mortality, major adverse cardiac events (MACEs) including arrhythmia, cardiac insufficiency and acute myocardial infarction (AMI), length of stay in an intensive care unit (ICU) and hospital. The exclusion criteria were as follows: (1) studies that involved participants who are < 18 years old; (2) studies that evaluated different interventions or did not include a reference group; (3) studies that did not report predefined outcomes or the data could not be extracted.

### Data Extraction and Risk of Bias Assessment

Two reviewers independently extracted the data using a predefined standardized form. The extracted data included first author, year of publication, sample size, patient characteristics, interventions, all clinical outcomes (the incidence of AKI, the need for RRT, all-cause mortality, MACEs including arrhythmia, cardiac insufficiency, and AMI, length of stay in ICU and length of stay in hospital). The risk of bias assessment was performed by two independent reviewers using the Cochrane risk of bias approach and a third reviewer was consulted if no consensus could be reached. The standard criteria included the following domains: random sequence generation, allocation concealment, blinding of participants and personnel, blinding of outcome assessment, incomplete outcome data, selective reporting, and other biases.

### Statistical Analysis

Outcomes were treated as dichotomous or continuous variables. We calculated risk ratios (RRs) with 95% confidence intervals (CIs) for dichotomous variables and mean differences (MDs) with 95% CIs for continuous variables. Heterogeneity among the studies was assessed by the *I*^2^ statistic, and when *I*^2^ was more than 50%, significant statistical heterogeneity was considered to be present (17). We used the fixed-effect model when the *I*^2^ values were < 25%. Otherwise, we used the random-effects model. Sensitivity analyses were conducted to test the robustness of the overall pooled effect. The presence of publication bias was evaluated by using a funnel plot. All comparisons were two-sided, and a *P* < 0.05 was considered statistically significant. If the mean or standard deviation of the outcomes could not be directly extracted from the studies, we estimated them from the sample size, median, range, and/or interquartile range (18, 19).

Review Manager (version 5.3, The Cochrane Collaboration, Oxford, United Kingdom) was used in all analyses.

### Trial Sequential Analysis

We conducted trial sequential analysis (TSA) in this meta-analysis to control the risk of random errors and assess whether the results were conclusive (20). Firm evidence for accepting or rejecting the anticipated intervention effect is considered clear and no further studies are needed if the cumulative Z-curve crossed the trial sequential monitoring boundary or entered the futility area. No conclusion is made and more studies are required to confirm the results if the Z-curve did not cross any of the boundaries or the required information size (RIS) has not been reached (21). In our meta-analysis, we performed the TSA with an overall risk of 5% of the type I error and estimated the RIS based on a RR reduction of 20% with a power of 80%. The control event rate was calculated according to the comparator group (22).

## Results

### Search Results and Study Characteristics

**Figure 1:**
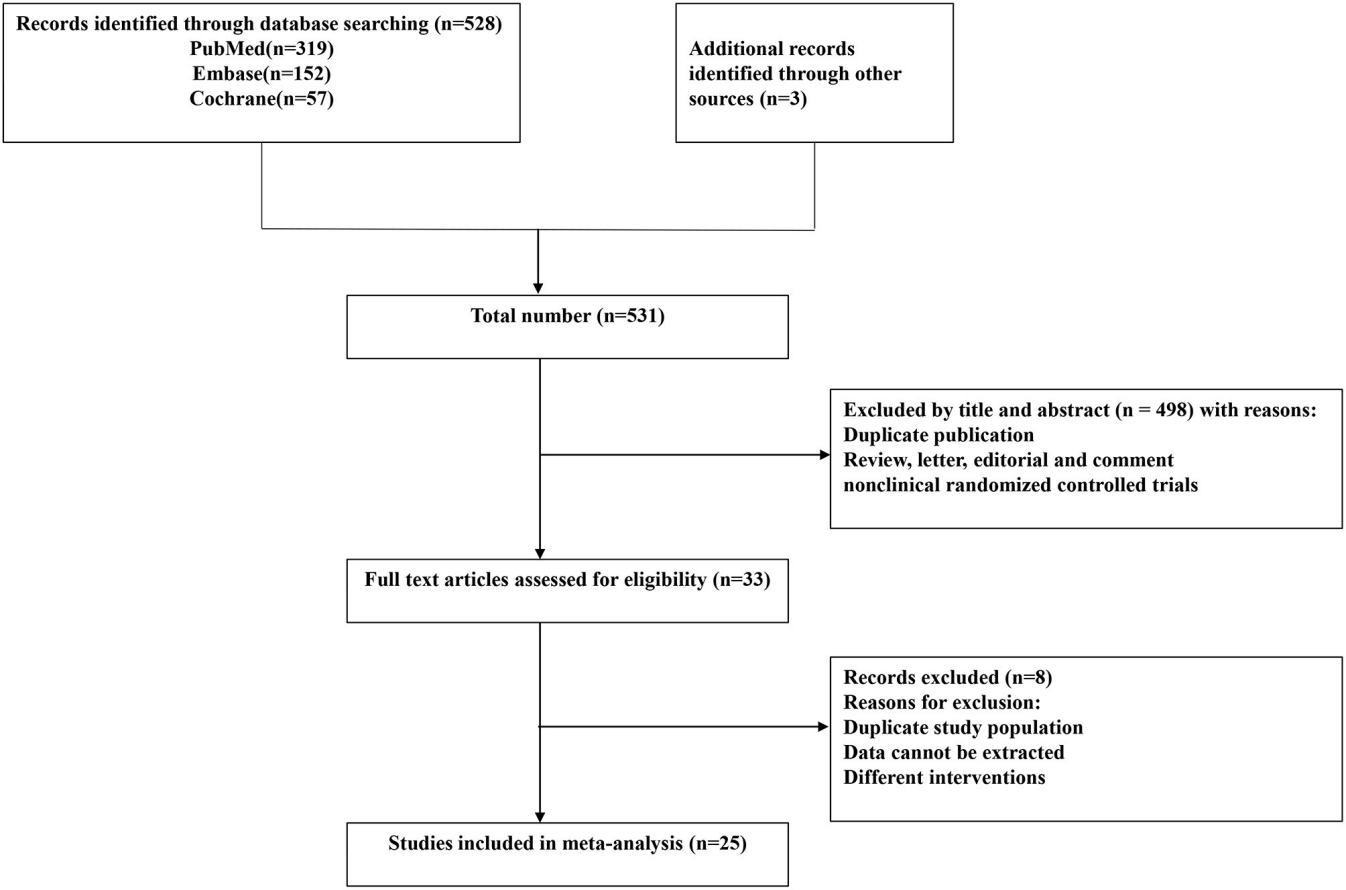
Preferred Reporting Items for Systematic Reviews and Meta-Analyses (PRISMA) flow diagram.

The flow chart describing the selection of the trials for this meta-analysis is presented in Figure 1. According to our search strategy, 531 potential studies were identified. After removing the duplicates and the studies that failed to meet the inclusion criteria, 33 studies were eligible for full-text reviews. Finally, only twenty-five (23–47) studies involving 2,444 patients were included in this meta-analysis. The characteristics of the included individual studies were summarized in Table 1. All included trials were reported between 2003 and 2018. The sample sizes of the included trials ranged from 20 to 295.

**Table 1:** Characteristics of included studies.

**Author year**	**Participants characteristics**	**Group**	**Number of participants (M/F)**	**Mean age, years**	**Intervention methods of NAC group**		**Definition of AKI in studies**	**Detection time point of AKI index**
Adabag (36)	Patients with CKD	NAC	50 (50/0)	70 ± 9^a^	PO	600 mg PO twice daily for a total of 14 doses	Serum creatinine>0.5 mg/dl or ≥25% increase from baseline	Postoperative days 5, 7, and 30
		Placebo	52 (52/0)	72 ± 9^a^				
Aldemir (31)	Geriatric patients (age > 65 years)	NAC	30 (18/12)	71.50 (69.00– 73.50) ^b^	IV	150 mg.kg^−1^ I.V. in 15 min after anesthesia induction, followed by 50 mg.kg^−1^.4h^−1^ and 100 mg.kg^−1^.16h^−1^. I.V.	Plasma creatinine levels of ≥1.5 mg/dl or >25% of baseline %	Postoperative 3 h, 12 h, day 1, day 2
		Placebo	30 (22/8)	70.50 (68.00– 73.25) ^b^				
Amini (29)	NYHA class of I-III	NAC	68 (41/27)	60.03 ± 10.37^a^	PO	600 mg PO twice daily from 24 h before the operation until two postoperative days	A serum Cr increase of by ≥ 0.3 mg/dl or ≥ 1.5 times baseline	48 h after surgery
		Standard of care	71 (49/22)	58.72 ± 8.57^a^				
Barr (24)	Patients with chronic renal insufficiency	NAC	20 (15/5)	73.8 ± 2.2^a^	PO	600 mg PO twice daily for a total of 4 doses	Creatinine clearances	Postoperative day 3
		Placebo	19 (13/6)	72.4 ± 2.0^a^				
Burns (46)	High-risk patients with age ≥ 70 years, diabetes mellitus, and more	NAC	148 (116/32)	68.9 ± 8.9^a^	IV	600 mg IV twice daily for a total of 4 doses	An absolute increase in serum creatinine level of > 0.5 mg/dl (44 μmol/L) or a 25% increase from baseline at any time	Within the first 5 postoperative days
		Placebo	147 (117/30)	69.2 ± 9.7^a^				
El-Hamamsy (33)	Low-risk patients underwent CABG with CPB	NAC	50 (43/7)	59.8 ± 7.8^a^	PO	600 mg PO 1 day before operation and 150 mg.kg^−1^ IV and 12.5 mg.kg^−1^.h^−1^ for 24 h IV	-	-
		Placebo	50 (46/4)	61.3 ± 7.4^a^				
Erdil (45)	Patients underwent CABG with CPB	NAC	50 (43/7)	59.8 ± 7.8^a^	PO	600 mg PO for 3 days before surgery and 300 mg *via* CPB prime	-	-
		Placebo	50 (46/4)	61.3 ± 7.4^a^				
Eren (44)	Low-risk patients underwent CABG with CPB (COPD patients excluded)	NAC	10 (8/2)	61.1 ± 4.8^a^	IV	100 mg.kg^−1^ IV 1 h before and 40 mg.kg^−1^.day^−1^ IV 24 h after CPB	-	-
		Placebo	10 (7/3)	60.5 ± 5.7^a^				
Fischer (35)	Low-risk patients underwent CABG with CPB	NAC	20 (12/8)	66.2 ± 11.8^a^	IV	100 mg.kg-1 in CPB prime and 20 mg.kg^−1^.h^−1^ until the end of CPB	-	-
		Placebo	20 (1/19)	66.5 ± 6.5^a^				
Haase (40)	High-risk patients with NYHA class III/IV, age>70 years, and more	NAC	30 (23/7)	68.9 ± 9.7^a^	IV	150 mg.kg^−1^ IV after anesthesia induction and 50 mg.kg^−1^ IV over 4 h and then 100 mg.kg^−1^ IV over 20 h	-	-
		Placebo	30 (21/9)	68.3 ± 9.3^a^				
Karahan (26)	Low-risk patients underwent CABG with CPB	NAC	21 (12/9)	58.6 ± 2.7^a^	Other	50 mg.kg-1 *via* cardioplegia	-	-
		Standard of care	23 (13/10)	56.4 ± 3.1^a^	-	-		
Kazemi (25)	Low-risk patients underwent CABG and /or valve with and without CPB	NAC	120 (91/29)	61.3 ± 9.8^a^	PO	1,200 mg PO twice daily from 48 h before and up to 72 h after surgery	-	-
		Placebo	120 (88/32)	58.2 ± 12.7^a^				
Kim (23)	Patients with an LVEF <40%	NAC	24 (21/3)	60.8 ± 8.4^a^	IV	100 mg.kg-1 IV after anesthesia induction and 40 mg.kg^−1^.day^−1^ IV for 24 h	An increase of creatinine to >2.0 mg/dl, or >50% increase in creatinine above the pre-operative baseline value	After surgery
		Placebo	24 (22/2)	65.3 ± 7.6^a^				
Koramaz (41)	Patients with coronary artery disease underwent CABG with CPB	NAC	15 (10/5)	60.2 ± 1.8^a^	Other	50 mg.kg-1 *via* cardioplegia	-	-
		Standard of care	15 (9/6)	57.5 ± 2.1^a^	-	-		
Orhan (32)	Low-risk patients underwent CABG with CPB	NAC	10 (7/3)	59.6 ± 5.48^a^	IV	50 mg.kg^−1^ IV	-	-
		Standard of care	10 (6/4)	61.8 ± 4.32^a^				
Ozaydin (39)	Low-risk patients underwent CABG and /or valve with and without CPB	NAC	58 (47/11)	57 ± 11^a^	IV	50 mg.kg^−1^ IV for 1 h before surgery and 50 mg.kg^−1^.24 h^−1^ IV for 48 h after surgery	-	-
		Placebo	57 (44/13)	59 ± 9^a^				
Prabhu (34)	Low-risk patients underwent CABG with CPB	NAC	28 (NR)	54.18 ± 9.89^a^	Other	50 mg.kg^−1^ *via* cardioplegia	-	-
		Standard of care	25 (NR)	53.04 ± 8.06^a^	-	-		
Prasad (30)	High-risk patients	NAC	35 (25/10)	55.60 ± 10.24^a^	PO	600 mg PO twice daily 1 day before surgery and 600 mg IV at anesthesia induction and then 600 mg PO twice daily until the second post-operative day	A postoperative increase in the serum creatinine level of more than 44 μmol l^∧^-1 (0.5 mg dl^∧^-1) or a rise in the creatinine level by 25% from the basal level	Preoperatively and postoperatively on the 1st, 2^nd^, and 5th day
		Standard of care	35 (28/7)	57.77 ± 9.36^a^				
Ristikankare (28)	Patients with chronic renal failure	NAC	38 (28/10)	72 (44–87)^b^	IV	150 mg.kg^−1^ IV after anesthesia induction and 50 mg.kg^−1^ IV over 4 h then 100 mg.kg^−1^ IV over 16 h	Increase of plasma creatinine over 25% from the baseline or an increase of more than 44 mmol liter^−1^.	The 1st, 3rd and 5th day after surgery
		Placebo	39 (34/5)	69 (51–81)^b^				
Santana-Santos (42)	Patients with CKD	NAC	35 (20/15)	65.0 ± 8.2^a^	IV	150 mg.kg^−1^ IV 2 h before surgery and 50 mg.kg^−1^ IV up to 6 h	Increase in serum creatinine of more than or equal to 0.3 mg/dl (≥ 26.4 μmol/l) or increase to more than 1.5-fold from baseline	In the first 72 h after surgery
		Placebo	35 (30/5)	64.0 ± 9.0^a^				
Sisillo (38)	Patients with chronic renal insufficiency	NAC	129 (65/64)	73 ± 6^a^	IV	1,200 mg IV before anesthesia induction and 3 boluses of 1,200 mg IV in 12 h intervals	An increase in serum creatinine concentration > 25% from baseline to the maximum value	The day before surgery, and every day for the following days
		Placebo	125 (60/65)	72 ± 6^a^				
Soleimani (27)	Low-risk patients underwent CABG with CPB	NAC	72 (39/33)	62.36 ± 8.85^a^	IV	50 mg.kg^−1^ IV in 30 min after anesthesia induction and 50 mg.kg^−1^ IV in 30 min for 2 days after surgery	-	-
		Placebo	69 (34/35)	60.7 ± 8.43^a^				
Song (43)	High-risk patients	NAC	57 (40/17)	68 ± 10^a^	IV	150 mg.kg^−1^ IV at anesthesia induction and 150 mg.kg^−1^ IV for 24 h	An increase in serum creatinine more than or equal to 0.3 mg/dl from baseline, or to 50% from baseline, or an oliguria <0.5 ml/kg per h for more than 6 h, within postoperative 48 h	Within postoperative 48 h
		Placebo	60 (43/17)	69 ± 8^a^				
Vento (37)	Patients underwent CABG with CPB	NAC	15 (15/0)	63.1 ± 1.9^a^	Other	100 mg.kg^−1^ *via* cardioplegia	-	-
		Standard of care	20 (20/0)	60.2 ± 1.7^a^	-	-		
Wijeysundera (47)	Patients with pre-existing moderate renal insufficiency	NAC	I: 88 (53/35)	74 ± 8^a^	IV	100 mg.kg^−1^ IV over 30 min after anesthesia induction and 20 mg.kg^−1^.h^−1^ IV until 4 h after CPB	A 72-h increase in creatinine concentration > 44 μmol·L−1 or 25%	Four weeks before surgery, and then daily for 72 h after surgery
		Placebo	87 (51/36)	73 ± 9^a^				

*C, control groups; CABG, coronary artery bypass graft; COPD, chronic obstructive pulmonary disease; CPB, cardiopulmonary bypass; EF, ejection fraction (cardiac); I, intervention groups; IV, intravenous; LVEF, left ventricular ejection fraction, NAC, N-acetylcysteine; NR, not reported; NYHA, New York heart association; PO, oral*.

*^a^Mean ± SD; ^b^Mean (range)*.

### Risk of Bias Assessment and Publication Bias of the Included Studies

**Figure 2:**
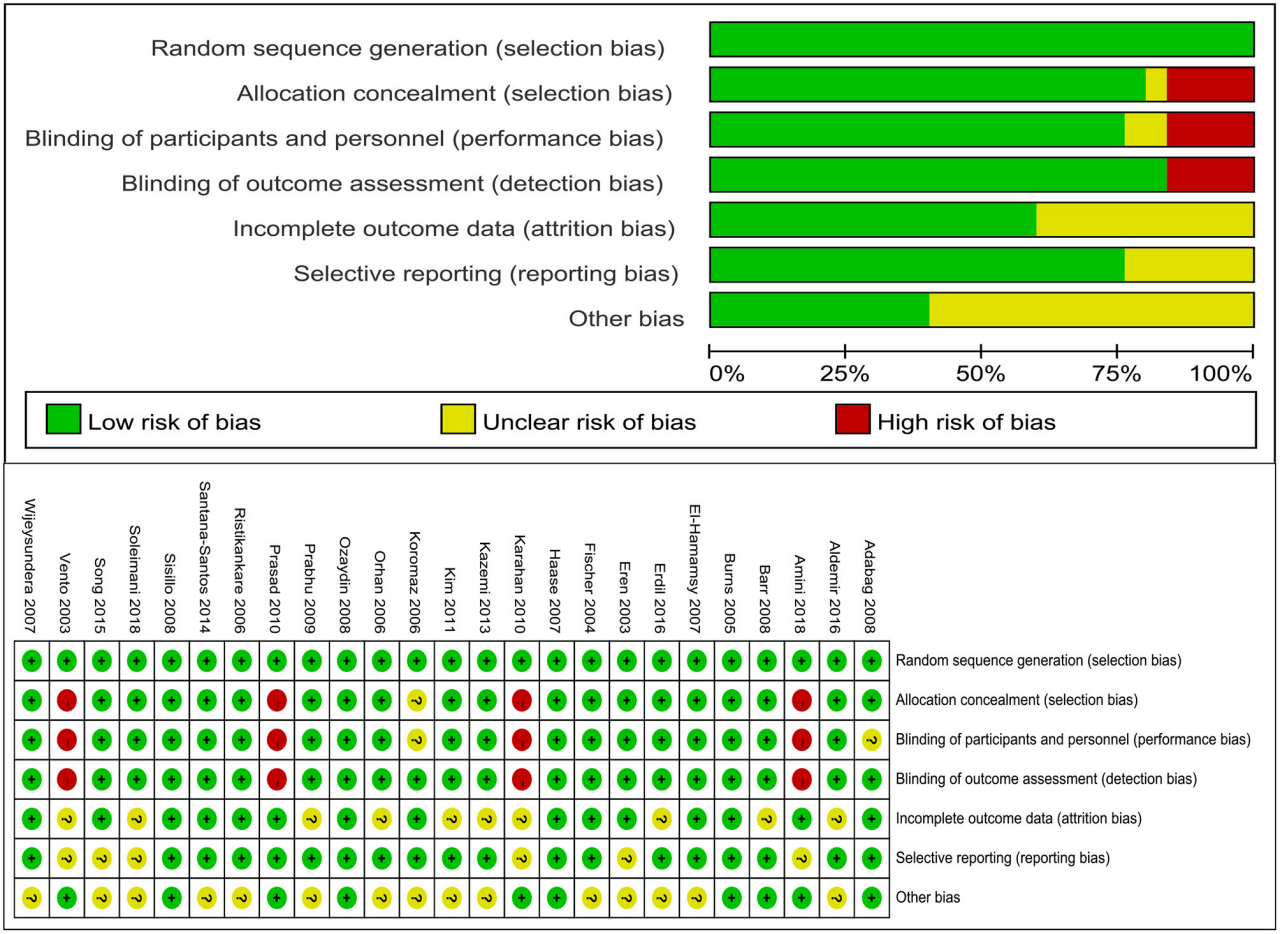
Risk of bias assessment. “+” indicates low risk of bias, “−” indicates high risk of bias, “?” indicates unclear risk of bias.

Review Manager 5.3 was used to assess the study quality in this study. A summary of the risk of bias in the included studies is presented in Figure 2. Random sequence generation was judged to be at a low risk of bias in all included studies. There is no significant publication bias of mortality (*P* = 0.652 for the Begg's test, *P* = 0.475 for the Egger's test), length of stay in ICU (*P* = 0.086 for the Begg's test, *P* = 0.163 for the Egger's test) and length of stay in hospital (*P* = 0.475 for the Begg's test, *P* = 0.181 for the Egger's test).

### The Incidence of AKI

**Figure 3:**
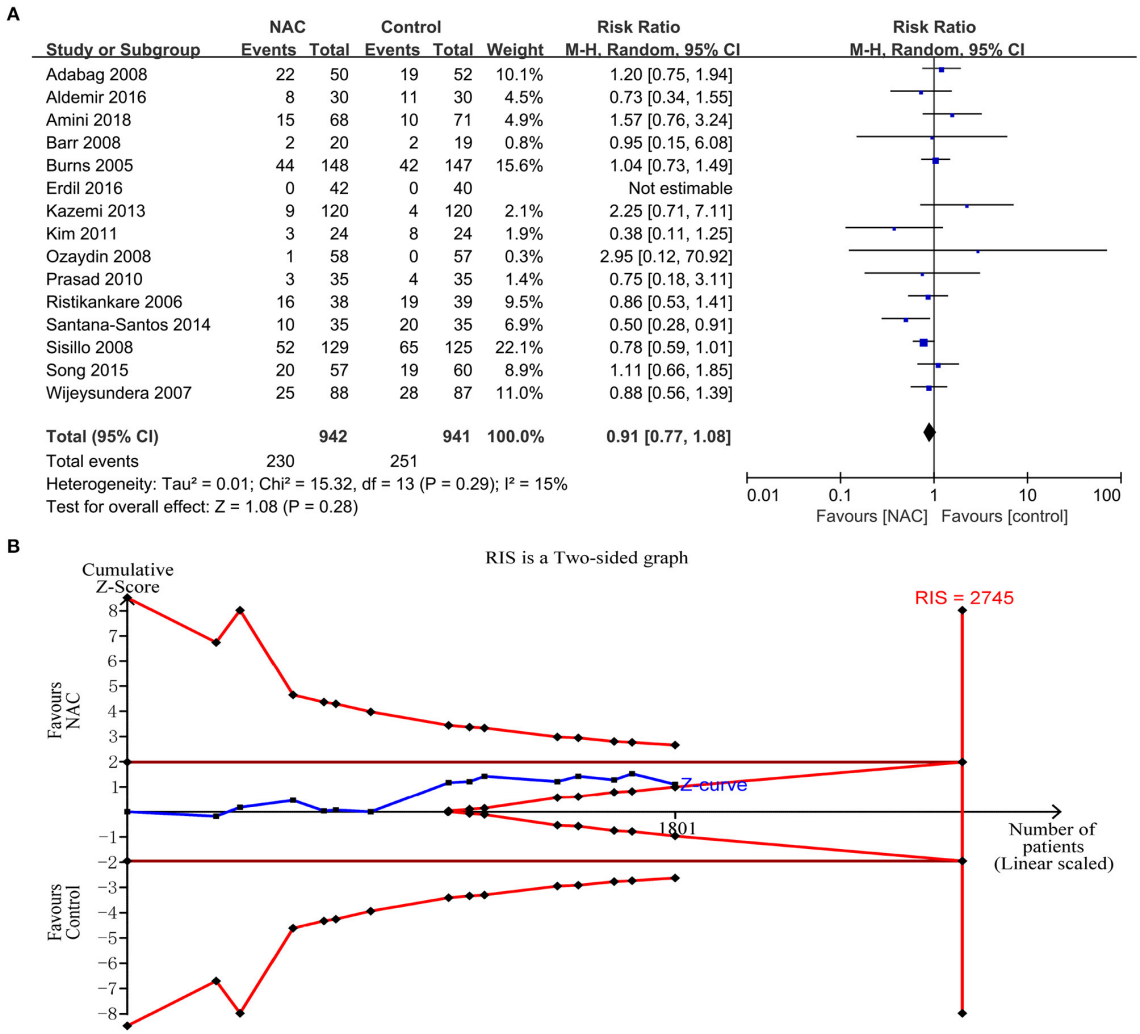
The effects of NAC on the incidence of AKI. **(A)** Forest plot of the incidence of AKI between NAC and controlled groups. **(B)** Fixed effects model of the TSA of the incidence of AKI between NAC and controlled groups. A diversity-adjusted information size of 2,745 participants was calculated based on a control event rate of 26.7% and a RR reduction of 20%, with α = 5% (two-sided), β = 20%, and *I*^2^ = 15%. The solid blue line represents the cumulative Z-curve, which did not cross the conventional boundary (solid dark red line), the trial sequential monitoring boundary (solid red line), and the futility boundary (solid red line).

Fifteen studies (23–25, 28–31, 36, 38, 39, 42, 43, 45–47) reported the incidence rate of AKI. The data from the trials showed that there was no significant difference in the incidence of AKI between the NAC groups and the controlled groups (RR = 0.91, 95%CI = 0.77, 1.08, *P* = 0.28, *I*^2^ = 15%) as shown in Figure 3A. However, the TSA could not confirm this result because the cumulative Z-curve did not cross the conventional boundary or the trial sequential monitoring boundary and did not cross the futility boundary (Figure 3B).

### The Need for RRT Among the Patients

**Figure 4:**
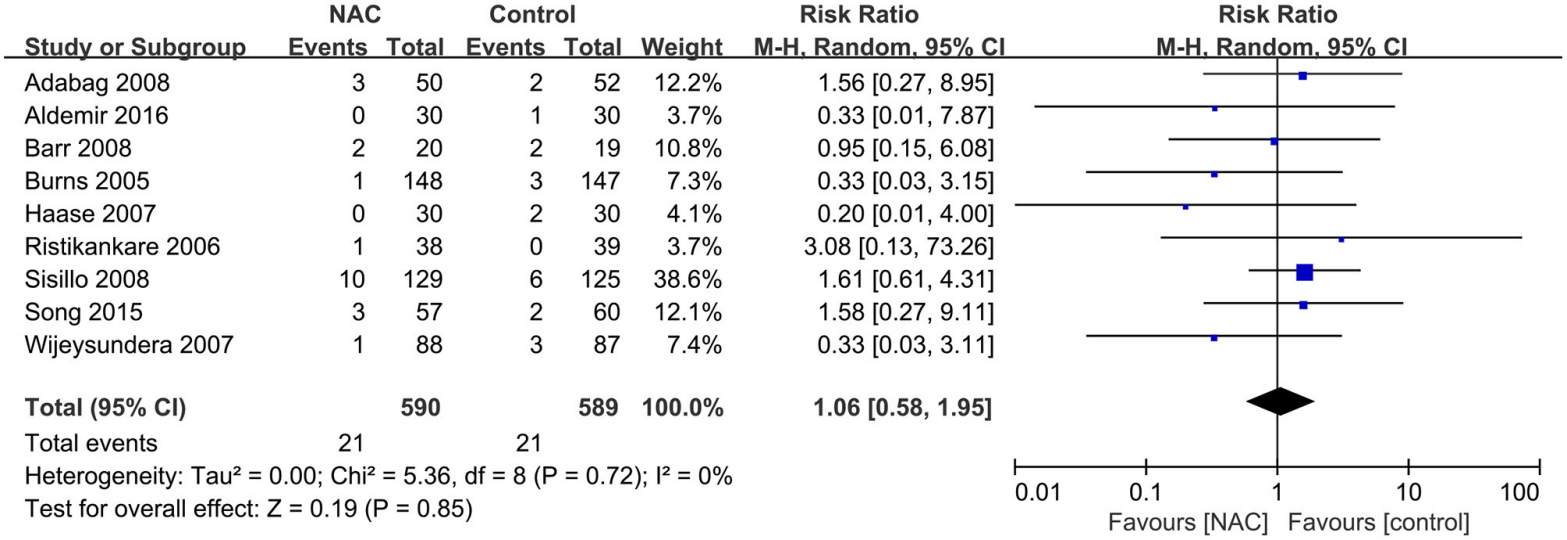
Forest plot of the effects of NAC on the need of patients for RRT after surgery.

Nine studies (24, 28, 31, 36, 38, 40, 43, 46, 47) with 1,179 patients reported the number of patients who required RRT among all included patients. No significant difference was found between the NAC and the controlled groups (RR = 1.06, 95% CI = 0.58, 1.95, *P* = 0.85, *I*^2^ = 0%) as depicted in Figure 4.

### All-Cause Mortality

**Figure 5:**
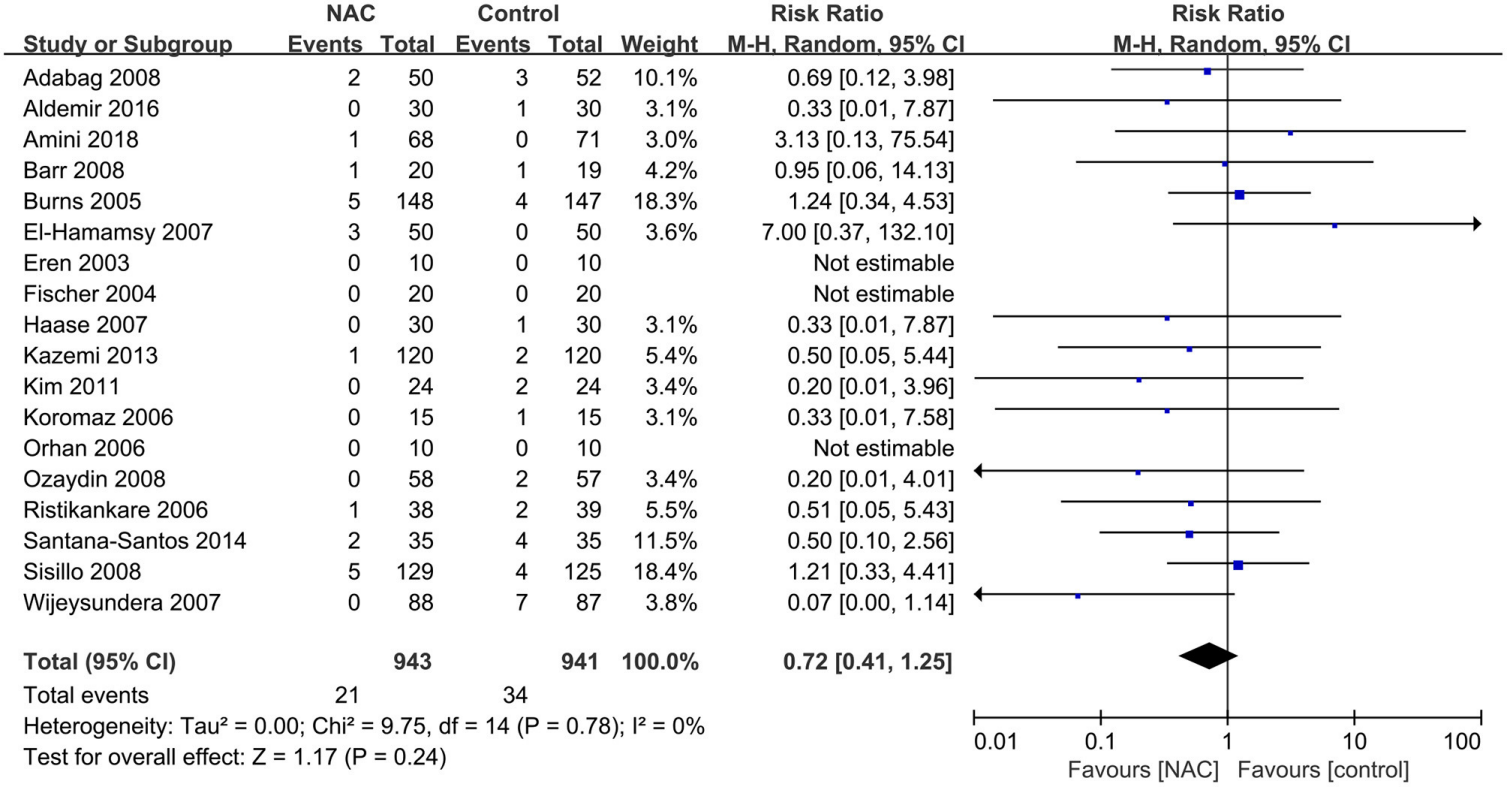
Forest plot of the effects of NAC on the incidence of all-cause mortality.

Eighteen (23–25, 28, 29, 31–33, 35, 36, 38–42, 44, 46, 47) of the included studies reported all-cause mortality. The mortality in the NAC group and the controlled group was 2.2 and 3.6%, respectively. The pooled results showed that the use of NAC could not reduce the risk of all-cause mortality compared with the use of placebo or just standard of care (RR = 0.72, 95% CI = 0.41, 1.25, *P* = 0.24, *I*^2^ = 0%) as presented in Figure 5.

### The Incidence of MACEs

**Figure 6:**
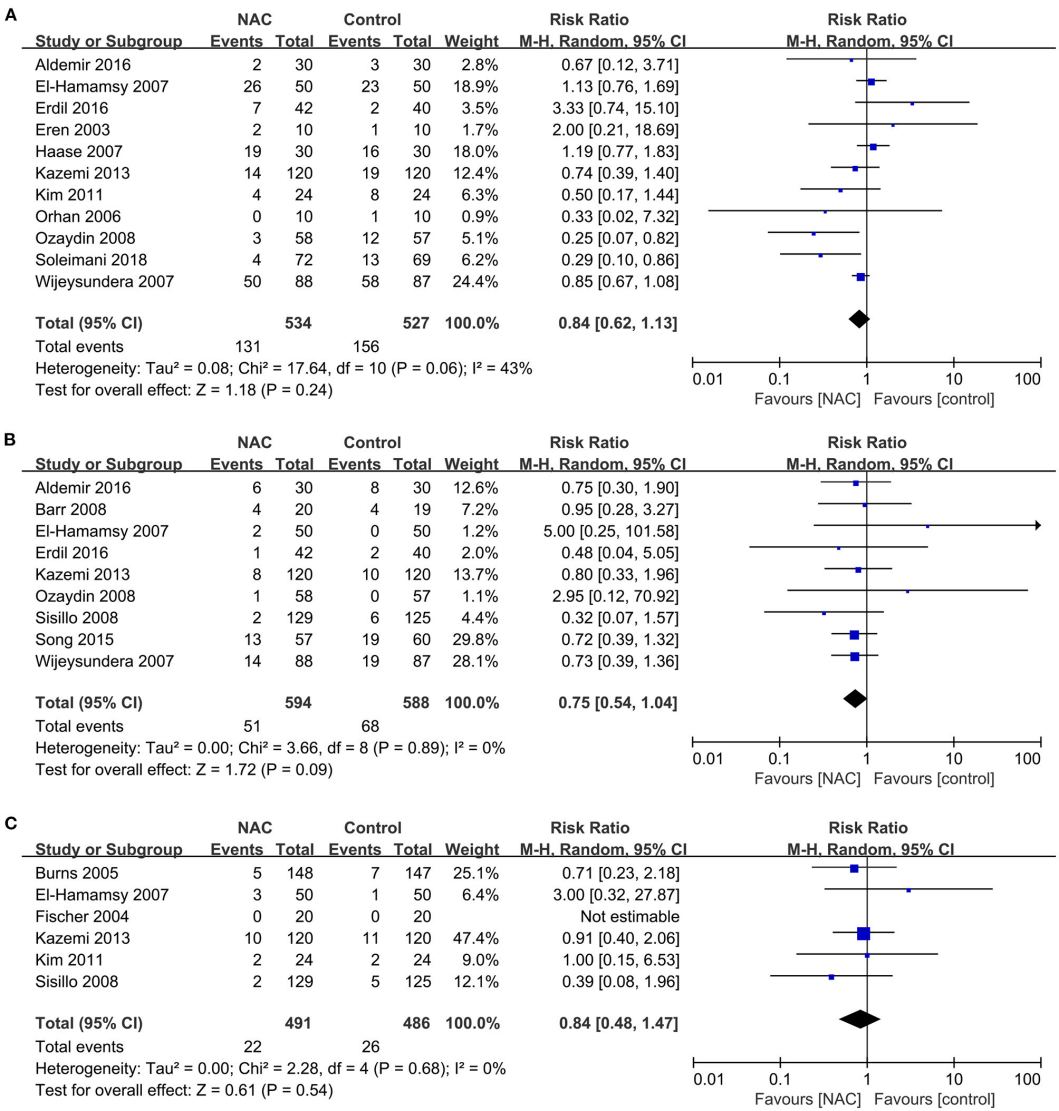
Forest plot of the effects of NAC on the incidence of MACEs. **(A)** Forest plot of arrhythmia. **(B)** Forest plot of cardiac insufficiency. **(C)** Forest plot of AMI.

The outcomes of MACEs analyzed in this study included arrhythmia, cardiac insufficiency and AMI. Eleven studies (23, 25, 27, 31–33, 39, 40, 44, 45, 47) reported the outcome of arrhythmia and the meta-analysis of these results shows that NAC treatment did not decrease the incidence of arrhythmia (RR = 0.84, 95% CI = 0.62, 1.13, *P* = 0.24, *I*^2^ = 43%) as illustrated in Figure 6A. Nine studies (24, 25, 31, 33, 38, 39, 43, 45, 47) and six studies (23, 25, 33, 35, 38, 46) reported cardiac insufficiency and AMI, respectively. The overall pooled analysis found no significant difference in the incidence of cardiac insufficiency and AMI between the NAC treatment and the controlled groups (RR = 0.75, 95% CI = 0.54, 1.04, *P* = 0.09, *I*^2^ = 0% and RR = 0.84, 95% CI = 0.48, 1.47, *P* = 0.54, *I*^2^ = 0%, respectively) as revealed in Figures 6B,C.

### Length of Stay in ICU and Hospital

**Figure 7:**
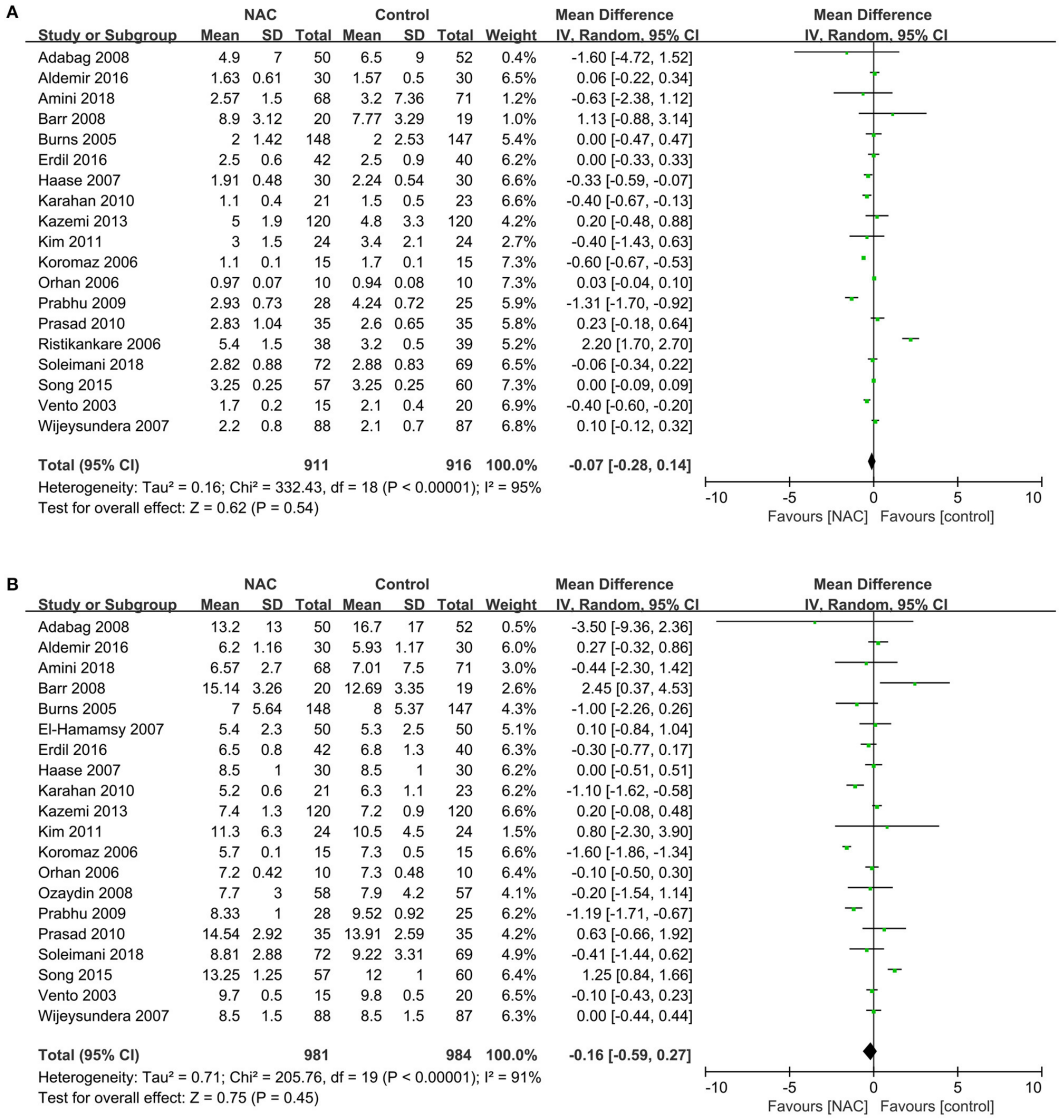
Forest plot of the effects of NAC on the length of stay in ICU and hospital. **(A)** Forest plot of the length of stay in ICU. **(B)** Forest plot of the length of stay in hospital.

Nineteen studies (23–32, 34, 36, 37, 40, 41, 43, 45–47) reported the length of stay in ICU in patients submitted to cardiac surgery. And twenty studies (23–27, 29–34, 36, 37, 39–41, 43, 45–47) reported the length of stay in hospital in patients. Results of these studies showed a non-statistically significant difference in the length of ICU and hospital stay between the NAC and controlled groups (MD = −0.07, 95% CI = −0.28, 0.14, P = 0.54, *I*^2^ = 95% and RR = −0.16, 95% CI = −0.59, 0.27, *P* = 0.45, *I*^2^ = 91%, respectively) as exhibited in Figures 7A,B.

### Effect of NAC Administration Methods on Outcomes

**Figure 8:**
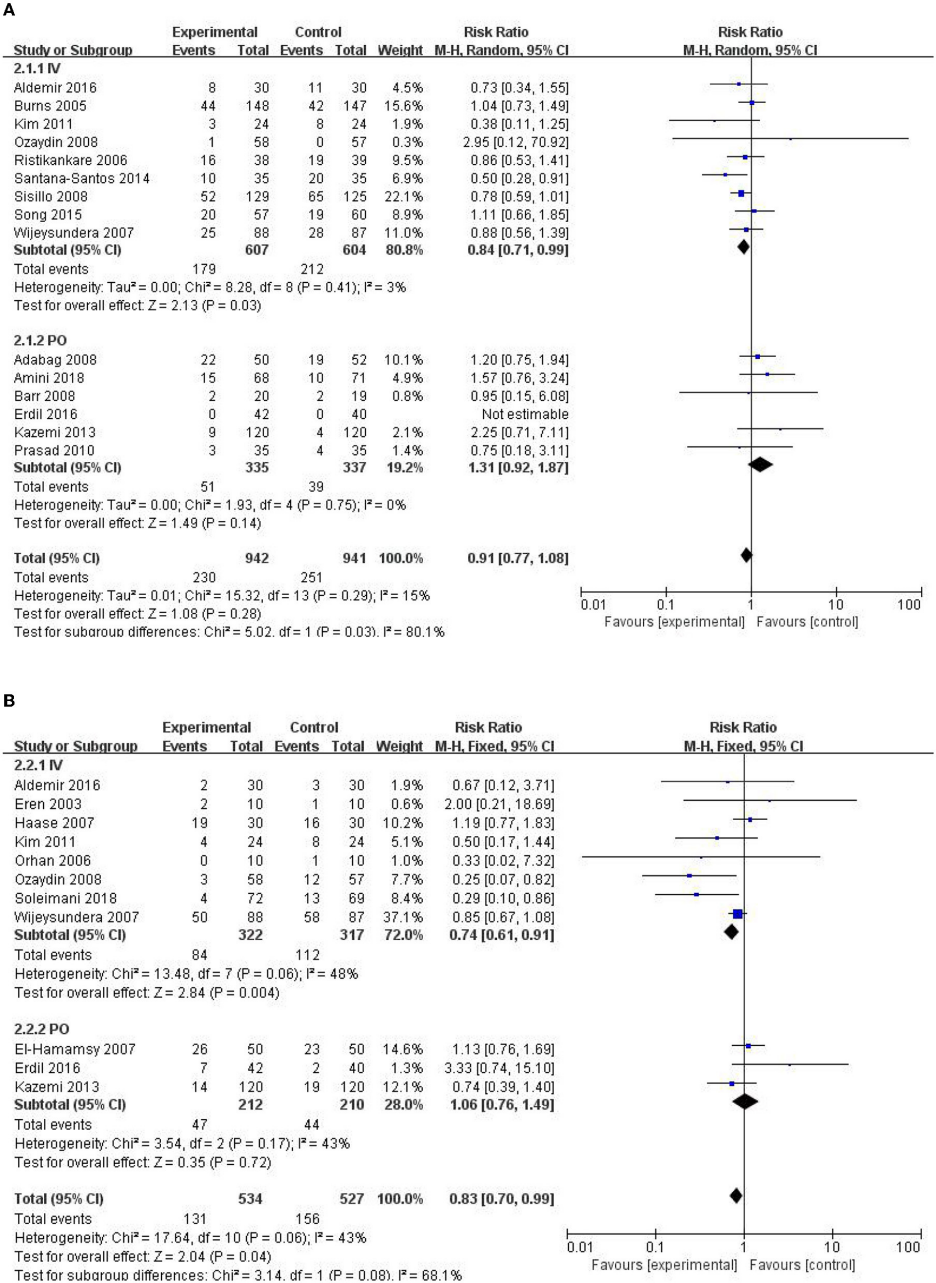
Forest plot of the effects of NAC administration methods on the incidence of AKI and arrhythmia. **(A)** Forest plot of the incidence of AKI. **(B)** Forest plot of the incidence of arrhythmia.

All the studies were divided according to NAC administration methods, and the effects on the outcome were analyzed respectively. The results showed that intravenous infusion instead of oral NAC could significantly reduce the incidence of AKI and arrhythmia (RR = 0.84, 95% CI = 0.71, 0.99, *P* = 0.03, *I*^2^ = 3% and RR = 0.74, 95% CI = 0.61, 0.91, *P* = 0.004, *I*^2^ = 48% respectively) as shown in Figures 8A,B. The addition of NAC to cardioplegia may reduce ICU and hospital stay, but significant statistical heterogeneity was observed. NAC has no significant effect on RRT, all-cause mortality, AMI, and cardiac insufficiency. Results of subgroup analysis showed administration methods of NAC had no significant effect on the need for RRT, all-cause mortality, AMI, and cardiac insufficiency among patients (Table 2).

**Table 2:** Effect of NAC administration methods on outcomes.

**Outcome**	**Administration methods**	**NO. of studies**	**Result**		**RR (95 CI%)**	**Heterogeneity *I*^2^ (*p*-value)**	**Z**	***p*-value**
			**NAC**	**Control**				
AKI	IV	23, 28, 31, 38, 39, 42, 43, 46, 47	179/607	212/604	0.84 (0.71, 0.99)	3% (0.41)	2.13	0.03
	PO	24, 25, 29, 30, 36, 45	51/335	39/337	1.31 (0.92, 1.37)	0% (0.75)	1.49	0.14
RRT	IV	28, 31, 38, 40, 43, 46, 47	490	488	1.07 (0.53, 2.19)	0% (0.46)	0.20	0.85
	PO	24, 36	100	101	1.03 (0.32, 3.35)	0% (0.70)	0.96	0.05
All-cause mortality	IV	23, 28, 31, 32, 35, 38, 39, 40, 42, 44, 46, 47	13/620	27/614	0.63 (0.33, 1.22)	0% (0.61)	1.36	0.17
	PO	24, 25, 29, 33, 36	8/308	6/312	1.11 (0.38, 3.27)	0% (0.61)	0.19	0.85
	cardioplegia	41	0/15	1/15	0.33 (0.01, 7.58)	-	0.69	0.49
Arrhythmia	IV	23, 27, 31, 32, 39, 40, 44, 47	84/322	112/317	0.74 (0.61, 0.91)	48% (0.06)	2.84	0.004
	PO	25, 33, 45	47/212	44/210	1.06 (0.76, 1.49)	43% (0.17)	0.35	0.72
Cardiac insufficiency	IV	31, 38, 39, 43	36/362	52/359	0.71 (0.49, 1.04)	0% (0.78)	1.77	0.08
	PO	24, 25, 33, 45	15/232	16/229	0.89 (0.45, 1.74)	0% (0.66)	0.35	0.72
AMI	IV	23, 35, 38, 46	17/417	23/412	0.75 (0.41, 1.38)	0% (0.65)	0.93	0.35
	PO	25, 33	5/74	3/74	1.58 (0.38, 6.63)	0% (0.53)	0.62	0.53
Length of stay in ICU	IV	23, 27, 28, 31, 32, 40, 43, 46, 47	497	496	0.16 (−0.19, 0.52)	86% (<0.001)	0.89	0.37
	PO	24, 25, 29, 30, 36, 45	335	337	0.02 (−0.13, 0.17)	0% (0.54)	0.25	0.80
	Cardioplegia	26, 34, 37, 41	79	83	−2.13 (−3.38, −0.88)	90% (<0.001)	3.34	0.0008
Length of stay in hospital	IV	23, 27, 31, 32, 39, 40, 43, 46, 47	517	514	0.10 (−0.36, 0.56)	76% (<0.001)	0.43	0.67
	PO	24, 25, 29, 30, 33, 36, 45	400	407	0.04 (−0.27, 0.36)	39% (0.12)	0.26	0.79
	Cardioplegia	26, 34, 37, 41	79	83	−2.13 (−3.38, −0.88)	90% (<0.001)	3.34	0.0008

## Discussion

Our meta-analysis showed some novel findings. The incidence of AKI in postoperative cardiac patients who received intravenous rather than oral NAC treatment was significantly lower than that in the control group. Intravenous injection of NAC can reduce the incidence of arrhythmia in patients after cardiac surgery while no difference was found in the incidence between intravenous injection and oral subgroups. Conversely, NAC has no significant effect on RRT, all-cause mortality, AMI, and cardiac insufficiency.

The intravenous NAC to patients with cardiac surgery is associated with a lower incidence of AKI, and the efficacy of oral NAC in preventing CI-AKI is inconclusive. Cardiac surgery-related acute renal injury (CSA-AKI) is the most common major complication of cardiac surgery (48). Although there is no consensus on the definition of post-cardiac surgery ARI, most of the fifteen studies stated that an increase in serum creatinine concentration > 25% from baseline is one of the conditions for the diagnosis of AKI. NAC has been shown to reduce the level of oxidative stress and reduce acute renal failure induced by ischemia-reperfusion. It can also improve renal aging and renal interstitial fibrosis through Sirtuin 1 activation and p53 deacetylation (49). There is no conclusive evidence supporting the benefit of the administration of NAC to prevent CSA-AKI in previous meta-analyses but our subgroup analysis reveals different results (12, 13). Of all the 15 studies that reported the incidence of AKI, 9 were administered intravenously with NAC, 6 were done orally. Results of a subgroup analysis according to the methods of administration show that the effect of NAC is related to the administration methods. Several possible mechanisms caused these differences such as dosage and duration. We listed the treatment protocol of NAC in each study in Table 1. The dosage of NAC by intravenous injection is often much larger than that by oral administration. For example, Aldemir gave patients 150 mg.kg^−1^ I.V. in 15 min after anesthesia induction, followed by 50 mg.kg^−1^.4h^−1^ and 100 mg.kg^−1^.16h^−1^. While the drug was administered at 600 mg PO twice daily for a total of 14 doses in Adabag's study. Furthermore, oral NAC was first absorbed through the digestive system, which may have a first-pass elimination effect, resulting in a decrease in blood concentration. It is worth mentioning that high-dose NAC is effective, but it has been also demonstrated to frequently cause adverse effects (50, 51). Before a large-scale application of NAC after cardiac surgery is recommended, further research is needed to explore the optimal dosage and method.

This meta-analysis shows that intravenous administration of NAC after cardiac surgery may reduce the incidence of arrhythmias. Baker and other studies found that the incidence of postoperative atrial fibrillation in the NAC group was 36% lower than that in the control group. But they did not explore the effect of drug use on the outcome (52). On the one hand, this confirms that NAC can significantly reduce the incidence of arrhythmias after cardiac surgery. On the other hand, there is a significant correlation between the occurrence of postoperative atrial fibrillation and the level of postoperative inflammation. NAC may reduce the occurrence of arrhythmias by inhibiting inflammatory storms.

In addition, we found that there was no significant difference in all-cause mortality, risk of AMI and cardiac insufficiency, rate of renal replacement therapy, and length of stay between the NAC group and the control group. This is consistent with previous studies (53). Given that we have included more RCT trials, the results may apply to a larger population.

Previous meta-analyses evaluating this topic have been published. However, there are several differences between the present study and the previous works. The present analysis includes the most RCT experiments representing the latest and most comprehensive study and we conducted a subgroup analysis of NAC for the first time based on the administration methods of NAC. Our meta-analysis also has some limitations. First, we only assessed the impact of NAC on the incidence of AKI but failed to assess the impact of the use of NAC on AKI to varying degrees because the vast majority of studies did not classify AKI or used different grading criteria. Second, a limitation is imposed on this meta-analysis because the relevant literatures included different degrees of differences such as the inclusion criteria of the study population and the dose and time of the use of NAC. These shortcomings may affect the results. Third, there is a lack of long-term follow-up results for patients, and a shortage of further subdivided MACEs, such as subgroup analysis of different types of arrhythmias.

## Conclusion

To sum up, this meta-analysis suggests that intravenous administration of NAC can reduce the incidence of AKI and arrhythmia in patients after cardiac surgery, but cannot reduce all-cause mortality, AMI, cardiac insufficiency, and the number of patients using RRT. Oral NAC has no significant effect on the outcomes of patients after cardiac surgery.

## Data Availability Statement

The original contributions presented in the study are included in the article/Supplementary Material, further inquiries can be directed to the corresponding author.

## Author Contributions

All authors listed have made a substantial, direct, and intellectual contribution to the work and approved it for publication.

## Conflict of Interest

The authors declare that the research was conducted in the absence of any commercial or financial relationships that could be construed as a potential conflict of interest.

## Publisher's Note

All claims expressed in this article are solely those of the authors and do not necessarily represent those of their affiliated organizations, or those of the publisher, the editors and the reviewers. Any product that may be evaluated in this article, or claim that may be made by its manufacturer, is not guaranteed or endorsed by the publisher.
